# Supplementary material for: In-vitro engineered human cerebral tissues mimic pathological circuit disturbances in 3D
Source: Commun Biol. 2022 Mar 23;5:254. doi: 10.1038/s42003-022-03203-4 (PMC8943047; doi:10.1038/s42003-022-03203-4)
Supplement: Supplementary file 2 — Supplementary information [file 42003_2022_3203_MOESM2_ESM.pdf]

Supplementary information

In-vitro engineered human cerebral tissues mimic pathological circuit disturbances in 3D

Aref Saberi[1,2]*, Albert P. Aldenkamp[3,4,5], Nicholas A. Kurniawan[1,2]*, and Carlijn V.C. Bouten[1,2]

[1]Department of Biomedical Engineering, Eindhoven University of Technology, Eindhoven, the Netherlands. [2]Institute for Complex Molecular Systems, Eindhoven, the Netherlands. [3]School for Mental Health and Neuroscience, Maastricht University Medical Center, Maastricht, the Netherlands. [4]Department of Behavioral Sciences, Epilepsy Center Kempenhaeghe, Heeze, the Netherlands. [5]Department of Electrical Engineering, Eindhoven University of Technology, Eindhoven, the Netherlands. *e-mail: a.saberi.aref@gmail.com and kurniawan@tue.nl

Supplementary Figures

Supplementary Figure 1 | Multiregional cerebral tissues generated by the MARC methodand STEMdiff cerebral organoids.

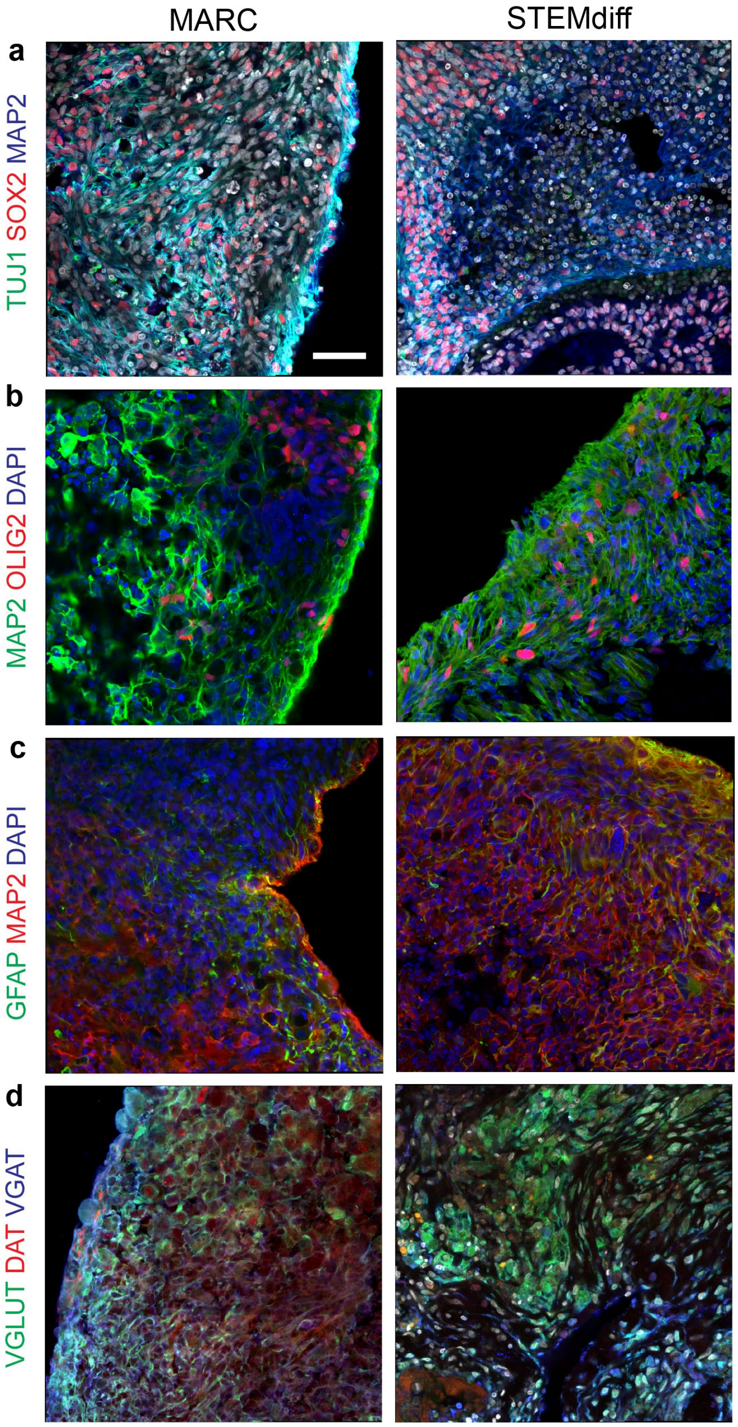

Immunohistochemical co-staining of multiple markers on 50-μm-thick sections of MARC-produced cerebral tissues (left column) and STEMdiff cerebral organoids (right column) at Day 90 showed no significant difference between the two protocols in the overall expression of distinct neuronal and neuroglial cell types. Co-staining of neural progenitor cells (NPCs; SOX2 in red), early neurons (Tuj1 in green) and mature neurons (MAP2 in blue) (**a**), co-staining of mature neurons (MAP2 in red), astrocyte marker (GFAP in green) and DNA marker (DAPI in blue) (**b**), mature oligodendrocytes marker (Olig2 in red), co-staining of mature neurons (MAP2 in green), and DNA marker (DAPI in blue) (**c**) and co-staining of dopaminergic neurons (DAT in red) and glutamatergic neurons (VGLUT1 in green) and GABAergic neurons (VGAT in blue) (**d**) (*n* = 5 samples across 2 independent experiments). Scale bar: 50 μm.

Supplementary Figure 2 | iS3CC chip: interacting but separated 3D co-culture chip.

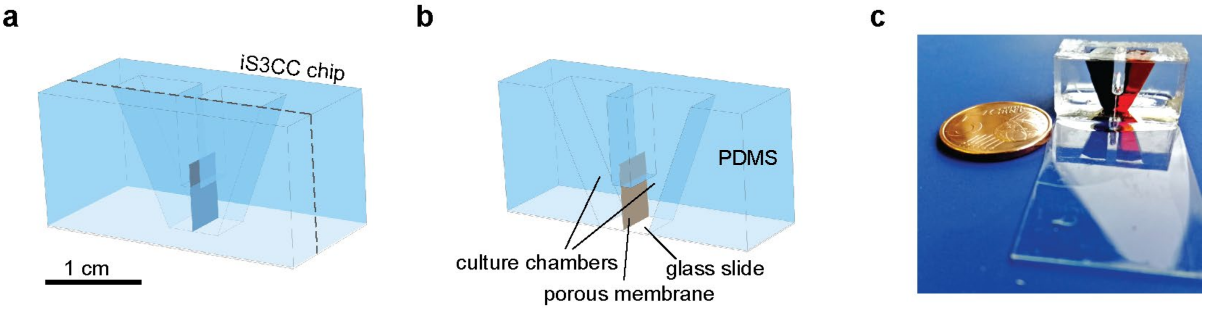

The design and features of the iS3CC chip are ideally suited for the formation of MARC-produced cerebral tissues and the investigations into the signal transmission between interconnected cerebral tissue cultures. **a**, A schematic illustration of the iS3CC chip. **b**, A cross-section view of the iS3CC chip including the most important features: the PDMS body, chambers wherein the cerebral tissues are cultured, the porous membrane, and the glass slide for microscopic visualization. The iS3CC chip consists of two symmetrically located right trapezoidal prism chambers with a height of 12 mm and a top opening of 6 mm by 4 mm and a bottom face of 2.5 mm by 4 mm. A 4 mm by 4 mm PET porous membrane has been vertically located between the two chambers allowing for three-dimensional connection along the z-axis between the chambers. The PDMS body and the porous membrane are immobilized on a thin microscopy glass slide, allowing for high resolution microscopy imaging. **c**, A photograph of an assembled iS3CC device where chambers are filled with red and blue dyes.

Supplementary Figure 3 | Formation and interconnection of MARC-produced cerebral tissues in the iS3CC chip.

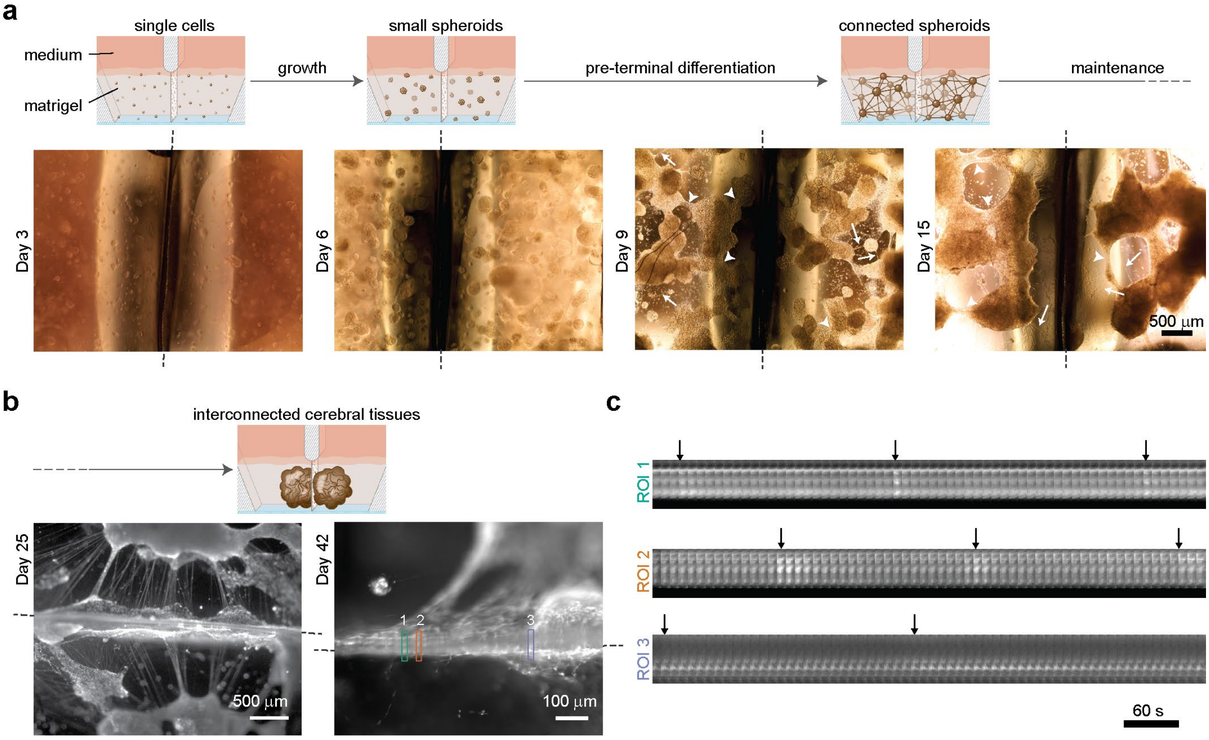

a–**b**, Progress of MARC culture in the iS3CC chip, resulting in interconnected cerebral tissues. Bottom-view phase-contrast images of both chambers of the iS3CC chip are shown at the bottom, demonstrating daily progress of MARC culture during different phases of cerebral tissue formation. White arrows indicate connective neurite outgrowths, whereas white arrowheads indicate merged neurite bundles between spheroids. Dashed lines indicate the porous membrane separating the chambers. Fluorescence pictures of intracellular calcium detected by fluo-4 direct, in cerebral tissues at day 25 and 42 and extended neurite outgrowths and bundles from both separated cultures, across the porous membrane indicated by horizontal dashed lines (**b**) (n = 30 samples across 6 independent experiments). **c**, Live calcium imaging in both interconnected cerebral tissues in the chambers of the iS3CC chip, demonstrating active connections between the separated cerebral tissues. See Supplementary Movie 2 for the corresponding time-lapse imaging data. The kymographs of three ROIs (shown in corresponding color code in **b**, right) show neural activity of connections

across the membrane as a function of time. Black arrows indicate the calcium transients during the imaging time. Scale bar: 60 s.

Supplementary Figure 4 | Penicillin G treatment induces abnormal excessive discharges.

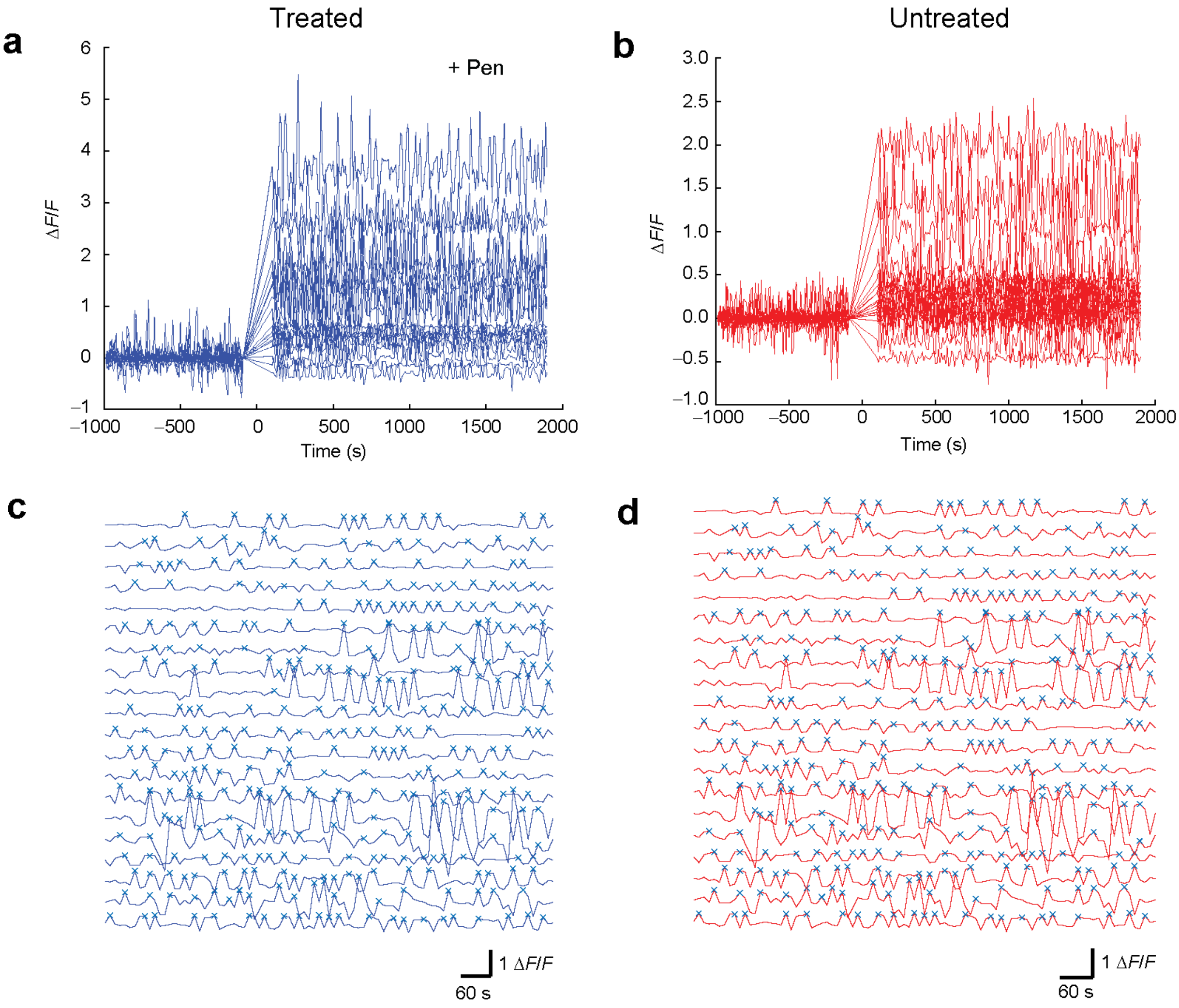

Time traces of normalized intensity (ΔF/F) from 20 representative neurons in the treated (**a**, blue) and untreated (**b**, red) tissues cultured in the two chambers of iS3CC chip are shown. Time 0 refers to the addition of Penicillin G ("Pen"). **c**–**d**, Zoom-in views of the pre-treatment time traces in the treated (**c**) and untreated (**d**) tissues. Data from each cell were offset for clarity. Blue crosses indicate detected transient spikes. Vertical scale bars: 1 ΔF/F. Horizontal scale bars: 60 s.

Supplementary Figure 5 | Immediate fluorescence increase in the treated chamber upon Penicillin G treatment.

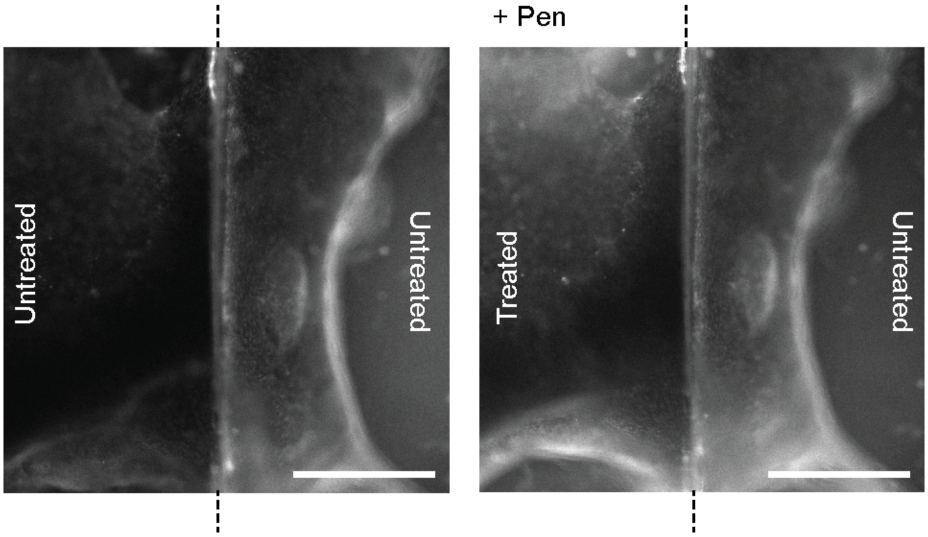

Two cerebral tissues were separately formed via MARC protocol in the two chambers of an iS3CC chip, separated by a membrane (black dashed line). One of the chambers (left, “treated”) was treated with Penicillin G (“Pen”), whereas the other (right, “untreated”) was not treated. Scale bar: 1mm.

Supplementary Figure 6 | Measurement of particle transfer between the chambers of the iS3CC chip across the porous membrane.

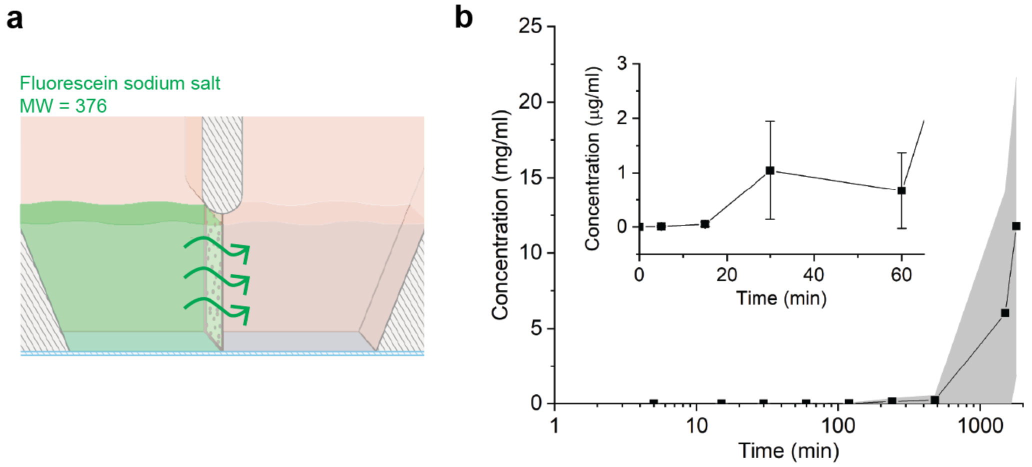

a, To simulate any potential diffusion between the two chambers, fluorescein sodium salt with comparable molecular weight (376.27 g/mol) to the Penicillin G sodium salt (367.37 g/mol) used in Figure 3 of the main text was added to one of the chambers of the iS3CC with the same final concentration as Penicillin treatment (100 mg/ml), whereas MilliQ water was added to the other chamber. The fluorescence of the solution in the latter chamber was measured over time using a plate reader and converted to concentration using a calibration curve. **b**, The measured concentration as a function of time (mean ± standard deviation, n = 6). Inset shows zoom-in of the first hour, i.e., the time-frame of the Penicillin-treatment seizure experiment in Figure 3. Error bars in the first 3 data points are smaller than the symbols. The data shows negligible diffusion across the membrane within this experimental time-frame. Furthermore, the diffusion of the fluorescent sodium salt takes place in pure MilliQ water and across a bare membrane in this diffusion experiment; the diffusion of Penicillin takes place in Matrigel and across a membrane covered by MARC tissues in the seizure experiment. Thus, we expect the former to be a gross overestimation of the diffusion rate in the Penicillin-treatment experiments. Given the negligible diffused concentrations within the experimental time-frame (60 min) of the Penicillin-treatment experiment, we believe that the observed propagation of the abnormal discharges occurs through the neuronal transmissions between the tissues.

Supplementary Figure 7 | Expression of the stress marker in MARC-produced cerebral tissues and STEMdiff cerebral organoids.

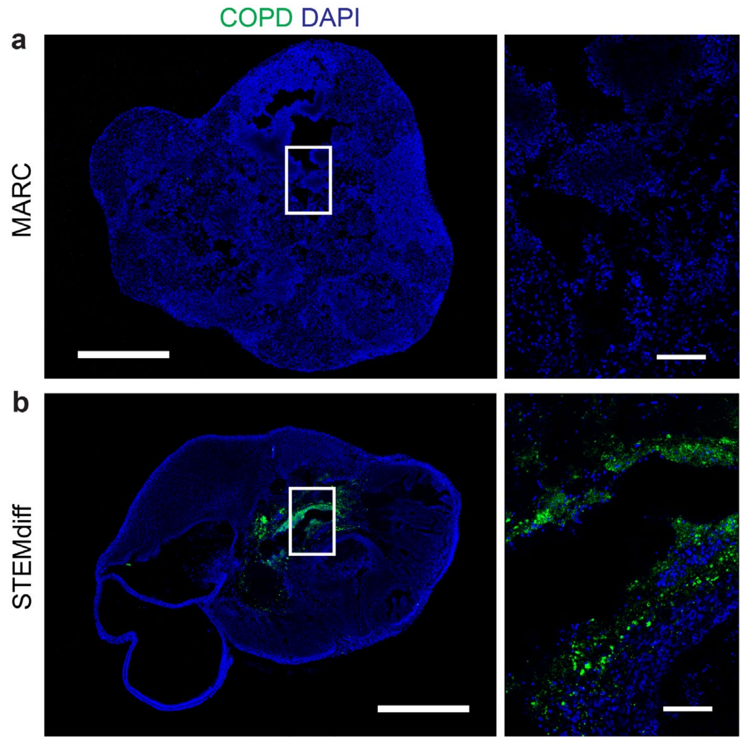

Immunohistochemical staining of the stress marker (COPD) in MARC-produced cerebral tissues (**a**) and STEMdiff cerebral organoids (**b**). The results indicate a higher level of COPD expression in the organoids compared to in the MARC-produced cerebral tissues. The right panel shows the zoom-in of the image in the left panel as indicated by the white squares (*n* = 5 samples across 2 independent experiments). Scale bar: 500 µm for the left panel and 50 µm for the right panel.
