# Supplementary material for: In-vitro engineered human cerebral tissues mimic pathological circuit disturbances in 3D
Source: Commun Biol. 2022 Mar 23;5:254. doi: 10.1038/s42003-022-03203-4 (PMC8943047; doi:10.1038/s42003-022-03203-4)
Supplement: Supplementary file 3 — Description of Additional Supplementary Files [file 42003_2022_3203_MOESM3_ESM.pdf]

Description of Additional Supplementary Files

File name: Supplementary Movie 1
Description: Time-lapse recording of live fluorescent calcium imaging of a MARC-produced cerebral tissue (week 4).

File name: Supplementary Movie 2
Description: Time-lapse recording of live fluorescent calcium imaging of neurite connections between two cerebral tissues across the porous membrane of the iS3CC (week 6).

File name: Supplementary Movie 3
Description: Time-lapse recording of live fluorescent calcium imaging of two interconnected cerebral tissues in the iS3CC before and after Penicillin treatment in the left chamber (week 6).

File name: Supplementary Software 1
Description: Matlab codes used to perform network analysis and synchrony of the neuron activities in the MARC tissues

File name: Supplementary Data 1
Description: Diffusion across chambers in iS3CC chip.xlsx. The measured concentration as a function of time in the receiving chamber.

File name: Supplementary Data 2
Description: Intensity and activity change upon Penicillin treatment.xlsx. Quantification of the change in fluorescence intensity and fold change in neuronal activity induced by addition of Penicillin G in the treated and untreated cerebral tissues. Each row represents each monitored cell.

File name: Supplementary Data 3
Description: Signal propagation traces.mat**.** Matlab data file containing structured data of the traces of signal propagation in both chambers in the Penicillin treatment experiment.

File name: Supplementary Data 4
Description: Neuronal network activity.mat. Matlab data file containing activity of neurons in the MARC tissue as reported through fluorescence intensity.
