# Supplementary material for: In-vitro engineered human cerebral tissues mimic pathological circuit disturbances in 3D
Source: Commun Biol. 2022 Mar 23;5:254. doi: 10.1038/s42003-022-03203-4 (PMC8943047; doi:10.1038/s42003-022-03203-4)
Supplement: Supplementary file 12 — Reporting Summary [file 42003_2022_3203_MOESM12_ESM.pdf]

nature research

Corresponding author(s): *Double-blind peer review submissions: write DBPR and your manuscript number here instead of author names.*

Last updated by author(s): *YYYY-MM-DD*

Reporting Summary

Nature Research wishes to improve the reproducibility of the work that we publish. This form provides structure for consistency and transparency in reporting. For further information on Nature Research policies, see Authors & Referees and the Editorial Policy Checklist.

Statistics

For all statistical analyses, confirm that the following items are present in the figure legend, table legend, main text, or Methods section.

n/a	Confirmed	
☐	☒	The exact sample size (*n*) for each experimental group/condition, given as a discrete number and unit of measurement
☐	☒	A statement on whether measurements were taken from distinct samples or whether the same sample was measured repeatedly
☒	☐	The statistical test(s) used AND whether they are one- or two-sided<br>*Only common tests should be described solely by name; describe more complex techniques in the Methods section.*
☒	☐	A description of all covariates tested
☒	☐	A description of any assumptions or corrections, such as tests of normality and adjustment for multiple comparisons
☒	☐	A full description of the statistical parameters including central tendency (e.g. means) or other basic estimates (e.g. regression coefficient) AND variation (e.g. standard deviation) or associated estimates of uncertainty (e.g. confidence intervals)
☒	☐	For null hypothesis testing, the test statistic (e.g. *F*, *t*, *r*) with confidence intervals, effect sizes, degrees of freedom and *P* value noted<br>*Give P values as exact values whenever suitable.*
☒	☐	For Bayesian analysis, information on the choice of priors and Markov chain Monte Carlo settings
☒	☐	For hierarchical and complex designs, identification of the appropriate level for tests and full reporting of outcomes
☒	☐	Estimates of effect sizes (e.g. Cohen's *d*, Pearson's *r*), indicating how they were calculated

Our web collection on statistics for biologists contains articles on many of the points above.

Software and code

Policy information about availability of computer code

Data collection	Calcium imaging data were obtained microscopically using Zeiss Zen (v2.3 Blue edition) software (Zeiss) or LAS X (version 3.7.0.20979 Leica Microsystems).
Data analysis	Calcium imaging data were analyzed with FIJI/ImageJ (version v1.52p), using Mosaic particle tracker plugin, and MATLAB (version R2018b, The Mathworks Inc.).

For manuscripts utilizing custom algorithms or software that are central to the research but not yet described in published literature, software must be made available to editors/reviewers. We strongly encourage code deposition in a community repository (e.g. GitHub). See the Nature Research guidelines for submitting code & software for further information.

Data

Policy information about availability of data

All manuscripts must include a data availability statement. This statement should provide the following information, where applicable:

- Accession codes, unique identifiers, or web links for publicly available datasets
- A list of figures that have associated raw data
- A description of any restrictions on data availability

The data of this study are available from the corresponding authors upon reasonable request.

Field-specific reporting

Please select the one below that is the best fit for your research. If you are not sure, read the appropriate sections before making your selection.

☒ Life sciences ☐ Behavioural & social sciences ☐ Ecological, evolutionary & environmental sciences

Life sciences study design

All studies must disclose on these points even when the disclosure is negative.

Sample size	Sample size for each experimental set ranges between 6 and 36.
Data exclusions	All data were included for analysis.
Replication	The complete culturing protocol was performed with 7 biological replicates. All replications were successful.
Randomization	Different samples and different batches were used for each type of experiment.
Blinding	No blinding was needed, since no statistical analysis was performed between the groups.

Reporting for specific materials, systems and methods

We require information from authors about some types of materials, experimental systems and methods used in many studies. Here, indicate whether each material, system or method listed is relevant to your study. If you are not sure if a list item applies to your research, read the appropriate section before selecting a response.

Materials & experimental systems

n/a	Involved in the study	
☐	☒	Antibodies
☐	☒	Eukaryotic cell lines
☒	☐	Palaeontology
☒	☐	Animals and other organisms
☒	☐	Human research participants
☒	☐	Clinical data

Methods

n/a	Involved in the study	
☒	☐	ChIP-seq
☒	☐	Flow cytometry
☒	☐	MRI-based neuroimaging

Antibodies

Antibodies used	Primary antibodies used were: rabbit polyclonal anti-Sox2 (1:250, Abcam, ab97959), mouse monoclonal anti-beta-Tubulin-III, Tuj1 (1:200, Merck, MAB1637), mouse monoclonal anti-vesicular glutamate transporter-I, VGLUT1 (1:250, Merck, AMAB91041), rat monoclonal anti-Dopamine Transporter, DAT (1:250, Abcam, ab5990), rabbit anti-VGAT (1:100, Merck, AB5062P), mouse anti-GFAP (1:200, Merck, G3893), chicken polyclonal anti-MAP2 (1:500, Abcam, ab5392), rabbit anti-OLIG2 (1:200, Merck, HPA003254) and mouse monoclonal anti-COPD (1:250, Invitrogen, MA5-18287).
Validation	Additional to elaborate description and tests reported in literature, all primary antibodies used in this study were positively validated with mouse brain slices.

Eukaryotic cell lines

Policy information about cell lines

Cell line source(s)	human induced pluripotent stem cells (hiPSCs)
Authentication	The cells were authenticated using RT-q-PCR as reported at DOI: 10.15252/embj.201695773.
Mycoplasma contamination	The cells tested negative on routinely performed (monthly) mycoplasma tests.
Commonly misidentified lines (See ICLAC register)	No commonly misidentified lines were used in this study.
